# BTK Inhibition Impairs the Innate Response Against Fungal Infection in Patients With Chronic Lymphocytic Leukemia

**DOI:** 10.3389/fimmu.2020.02158

**Published:** 2020-08-28

**Authors:** Stefania Fiorcari, Rossana Maffei, Daniela Vallerini, Lydia Scarfò, Patrizia Barozzi, Monica Maccaferri, Leonardo Potenza, Paolo Ghia, Mario Luppi, Roberto Marasca

**Affiliations:** ^1^Hematology Unit, Department of Medical and Surgical Sciences, University of Modena and Reggio Emilia, Modena, Italy; ^2^Hematology Unit, Department of Oncology and Hematology, A.O.U of Modena, Policlinico, Modena, Italy; ^3^Università Vita-Salute San Raffaele and IRCCS Istituto Scientifico San Raffaele, Milan, Italy

**Keywords:** chronic lymphocytic le, ibrutinib, macrophages, fungal infection, immunomodulation

## Abstract

Infections represent a cause of morbidity and mortality in patients affected by chronic lymphocytic leukemia (CLL). Introduction of new drugs in CLL clinical practice has showed impressive efficacy, in particular those targeting BTK. Among the consistent clinical data, an increasing number of reports describing the occurrence of unexpected opportunistic fungal infections has been reported during treatment with ibrutinib in the first 6 months of treatment. The reason underlying manifestations of invasive fungal infections in patients treated with ibrutinib is still under investigation. Our study aimed to understand the impact of BTK inhibition on immune response to fungal infection mediated by macrophages and CD14+ monocytic population obtained from CLL patients. Exposure to ibrutinib and acalabrutinib reduced signaling pathways activated by *Aspergillus fumigatus* determining an exacerbation of an immunosuppressive signature, a reduction of phagocytosis and a significant deficit in the secretion of inflammatory cytokines either in macrophages and monocytes isolated from CLL patients and healthy donors. These effects lead to a failure in completely counteracting conidia germination. In addition we investigated the biological effects of ibrutinib on monocyte counterpart in patients who were undergoing therapy. A significant impairment in cytokine secretion and a deficit of phagocytosis in circulating monocytes were detected after 3 months of treatment. Thus, our results uncover modifications in the innate response in CLL patients induced by ibrutinib that may impair the immunological response to fungal infection.

**KEYPOINTS:**

• BTK inhibition affects a productive immune response of CLL-associated macrophages (NLC) during *Aspergillus fumigatus* infection.
• Reduction of TNF-α secretion and phagocytosis are detected in monocytes isolated from CLL patients during ibrutinib therapy.

## Introduction

Fungi are associated with a wide spectrum of diseases in humans ranging from acute pulmonary manifestations and cutaneous lesions in immunocompetent individuals to severe infections in immunocompromised patients. The immune system does not stay unarmed against fungi and acts with pro- and anti-inflammatory signals to eradicate the infection. Infections by *Aspergillus fumigatus* (*A. fumigatus*) from surrounding environment are very common and the immunocompetent population is able to annihilate it. The immunosuppressed populations, caused particularly by cancer, chemotherapy, organ transplantation and autoimmune disease, are at risk to develop invasive aspergillosis (IA) (1). In invasive disease, Aspergillus spores germinate into filamentous hyphae and invade destroying tissue and organs with a 50–60% of mortality (2). Protection from infections includes recognition of fungi, activation of host immunity and killing of spores to suppress fungal dissemination. Macrophages recognize microbes and secrete cytokines and chemokines recruiting and activating other cell subsets in order to obstruct it. During an *A. fumigatus* infection, macrophages target and phagocytose conidia with pseudopods and endocytose conidia in an actin-dependent manner. Moreover, macrophages play a sentinel role secreting pro-inflammatory cytokines as TNF-α, IL-6, and IL-18 (3).

Infections represent a leading cause of morbidity and mortality in patients affected by chronic lymphocytic leukemia (CLL). CLL patients experience increased risk for infections related to deficits in cell-mediated immunity, such as hypogammaglobulinemia, dysfunctions of T-cell population and defects in antibody dependent cellular cytotoxicity. CLL cells are not innocent bystanders, but escape immunosurveillance and shape the surrounding microenvironment to “pull the strings” of immune effector cells (4, 5). The CLL-derived monocytic population presents altered properties mainly related to a reduced expression of genes involved in inflammation and phagocytosis, that contribute to an exacerbation of immunosuppressive features (6). CD14+ monocytes differentiate *in vitro* into large, round and adherent cells called nurse-like cells (NLC). Direct contact between NLC and CLL cells contribute to leukemic cells survival and protection from drug-induced apoptosis. Phenotypically, NLC are considered CLL-specific M2-skewed tumor associated macrophages characterized by high CD11b, CD163, CD206, HLA-DR, HGF, and IDO expression and by dysregulation of genes involved in immunocompetence (7–10). A recent evidence has shown that NLC express Bruton tyrosine kinase (BTK), a cytoplasmic tyrosine kinase belonging to Tec family kinases. The relevance of BTK expression in macrophages is related to regulation of macrophage lineage commitment. BTK is involved in the induction of signals driving M1 polarization, but it also functions as a negative regulator of M2-polarizing signaling pathways mediating host immunity against fungal pathogens (11). Fungal pathogens activate toll-like receptors (TLRs) initiating downstream signaling including BTK that promotes innate immune responses. Stimulation of TLR9, that leads to activation of BTK during *A. fumigatus* infection in macrophages, is required for an inflammatory response (12, 13).

Ibrutinib is a first-in-class potent inhibitor of BTK that binds covalently to Cys-481 in the ATP-binding domain of the kinase. Despite the consistent impressive clinical data, some patients with CLL show limited benefit from BTK inhibition due to discontinuation caused by adverse events. Of note, in the last years several reports have described the occurrence of opportunistic fungal infections in ibrutinib-treated patients. Different studies have shown association between ibrutinib and IA (14–17). Although ibrutinib has shown excellent effects on CLL cell component inducing mobilization of lymphocytes from tissue into the blood with the consequent cell death, recently different studies have demonstrated the on-target effects on off-tumor cells related to tumor microenvironment. In particular, ibrutinib targets BTK expressed by NLC exacerbating the immunosuppressive profile and probably supporting the protection of residual CLL cells inside tissue niches (11, 18, 19). Acalabrutinib is a potent highly selective BTK inhibitor, FDA-approved for the treatment of CLL, with different rate of infections in preliminary reports (20–22).

Considering the relevance of understanding the mechanism by which BTK inhibition impairs immune response, we sought to determine the effects of ibrutinib and acalabrutinib on the macrophage/monocyte population in CLL during a fungal infection by *A. fumigatus.* We demonstrate that BTK inhibition upon exposure to ibrutinib and acalabrutinib significantly hampered the inflammatory response of NLC during *A. fumigatus* infection. Clinically, our results are supported by a comprehensive analysis on the CD14+ monocytic population in CLL patients who were undergoing ibrutinib therapy. We defined the effects of ibrutinib in CLL patients who undergoing ibrutinib therapy supporting previous clinical observations showing attenuation of a productive immunological response affecting the inflammatory profile of CLL monocytes during *A. fumigatus* infection.

## Materials and Methods

### Patients and Samples

Blood samples from patients that matched standard diagnostic criteria for CLL were obtained from the Hematology Unit of Modena Hospital, Italy with a protocol approved by the local Institutional Review Board. Peripheral blood mononuclear cells (PBMCs) were isolated by Ficoll density gradient centrifugation and used fresh or cryopreserved in RPMI-1640 medium (Life Technologies, Carlsbad, CA, United States), 50% fetal bovine serum (FBS), and 10% dimethyl sulfoxide (DMSO) and stored in liquid nitrogen until use. To generate NLC, PBMCs from CLL patients were cultured (10^7^/mL) in RPMI-1640 medium with 10% FBS, 50 μg/mL gentamicin, 100 U/mL penicillin, 100 μg/mL streptomycin for 15 days. Fresh medium was added to the culture every 3 days. NLC were generated as previously described (7). NLC were treated with ibrutinib or acalabrutinib (1 μM) or vehicle before evaluations (23, 24).

### Analysis of Gene Expression Profile

Chronic lymphocytic leukemia cells were carefully washed off and adherent NLC were treated over-night with 1 μM ibrutinib or vehicle. NLC were lysed to obtain RNA samples. Total RNA was extracted by using RNeasy Mini kit Plus (QIAGEN). Large-scale gene expression profiling (GEP) was performed by hybridizing RNA on 4 × 44K Whole Human Genome Microarray (Agilent Technologies). Fluorescence data were analyzed with Feature Extraction Software v10.5 (Agilent Technologies). Supervised analysis based on paired *t*-test with multiple testing correction (Benjamini Hochberg FDR) were performed by using Gene Spring GX v11.5 (Agilent) software. Genes were defined as differentially expressed between ibrutinib-treated vs. vehicle-treated group at a significant level of *P* < 0.05 and with a fold change cut off ±2. Gene Ontology Tool^1^ was used to classify genes in functional categories. GO offers a comprehensive analysis from molecular level to larger pathways deepening and understanding the gene functions. Data have been deposited in NCBIs Gene Expression Omnibus (GEO^2^, GSE142292).

### Real-Time PCR

RNA was extracted with the RNeasy Plus Mini kit (Qiagen, Valencia, CA, United States). RNA (100 ng) was reverse transcribed using Transcription High fidelity cDNA Synthesis kit (Roche Applied Science, Penzeberg, Germany). All samples were analyzed in real-time on LightCycler 480v.2 (Roche) in duplicate). Amplification of the sequence of interest was normalized to an housekeeping reference gene (Glyceraldehyde 3-phosphate dehydrogenase, GAPDH) and compared to a calibrator sample (Universal Human Reference RNA; Stratagene, Cedar Creek, TX, United States). Primers are listed in Supplementary Table 1.

### Human Magnetic Luminex Screening Assay

Nurse-like cells were treated overnight with ibrutinib or DMSO and the following day conditioned media were collected, centrifuged to pellet residual cells and store at −80°C. Amount of CCL3, CCL4, CCL18, CCL22, CXCL10, CXCL9, CXCL12, CXCL13, IL-12, IL-2, IL-8 were measured in duplicate in conditioned medium by laboratory service through luminex screening assay (Labospace, Italy).

### XTT Assay

Nurse-like cells were cultured in 96 well plate in triplicate and then treated with ibrutinib or DMSO. Conidia of *A. fumigatus* (2 × 10^3^ per well) were plated over NLC or alone (positive control) and incubated at 37°C for 36 h to allow germination. A conidia vs. NLC ratio of 1:100 was used. Each experimental condition was performed in triplicate. To characterize the lytic activity of NLC against *A. fumigatus*, a colorimetric assay with [2,3-bis(2-methoxy-4-nitro-5-sulfophenyl)2H-tetrazolium-5-carboxyanilide] sodium salt (XTT; Sigma) plus coenzyme Q0 (2,3-dimethoxy-5-methyl-1,4-benzoquinone; Sigma) was used. Anti-hyphal activity was expressed normalizing the absorbance of experimental wells (CTRL vs. ibrutinib) with NLC to the absorbance of wells with hyphae only ^∗^ 100.

### NLC Phagocytosis Assay

Phagocytosis was inspected by using CytoSelect^TM^ 96-Well Phagocytosis assay (Cell Biolabs, San Diego, CA, United States) according to manufacturer’s instructions. NLC were generated from CLL patients and then treated with ibrutinib for 1 h. After incubation, zymosan particles were added to cells for 1 h, then NLC were fixed and external zymosan particles were blocked. After permeabilization, zymosan particles engulfed by NLC were measured by colorimetric detection.

### CD14+ Monocytes Phagocytosis Assay

Peripheral blood mononuclear cells isolated from CLL patients or healthy donors (HD) were suspended in culture medium either in presence or absence of ibrutinib for 1 h. Zymosan A FITC-fluorescent BioParticles (Molecular probes, Eugene, Oregon) were added and incubated with cells at 37°C for 1 h. Then, PBMCs were stained with CD14 APC and CD11b PE Abs. To distinguish the cells which have phagocytosed these from those simply binding the beads at the surface, a short incubation with trypan blue, followed by a wash with PBS, quenched surface FITC fluorescence. Analysis was performed by flow cytometry gating CD14+/CD11b+ cells and analyzing the mean fluorescence intensity in the positive zymosan population.

### Cytokine Secretion Assay

Nurse-like cells or Peripheral blood mononuclear cells isolated from CLL patients or HD were treated with ibrutinib or acalabrutinib for 24 h and stimulated with germinated boiled killed *A. fumigatus* inactivated conidia or zymosan and analyzed using cytokine secretion assay (CSA) for TNF-α according to manufacturer’s instructions (CSA Detection kit; Miltenyi Biotec). Cells were immunostained with TNF-α catch reagent and incubated for 2 h at 37°C to allow cytokine secretion. After washes, cells were labeled with TNF-α Detection antibody conjugated to PE. To identify the monocytic population, PBMCs were stained with CD14 APC antibody. An isotype control sample for each condition was acquired to exclude autofluorescence background.

### Immunoblotting

Nurse-like cells were pretreated with ibrutinib or acalabrutinib overnight following to stimulation with 2 × 10^5^/ml germinated boiled killed *A. fumigatus* inactivated conidia for 2 h or 50 μg/ml zymosan for 1h. Proteins (80 μg/lane) were electrophoresed on 4–20% SDS-polyacrylamide gradient gels (Bio-Rad Laboratories, Hercules, CA, United States). Membranes were immunoblotted with primary antibodies listed in Supplementary Table 2 and incubated with species-specific horseradish peroxidase (HRP)-conjugated secondary antibody (diluted 1:50,000; GE Healthcare, Uppsala, Sweden) for 1 h and developed using HRP conjugates Western Bright Sirius (Advasta, Menlo Park, CA, United States). Images were acquired and analyzed using Image Lab Software v.3.0 (Biorad Laboratories).

### *In vivo* Analysis

Peripheral blood mononuclear cells from CLL patients under ibrutinib therapy were collected and stored in liquid nitrogen pre and after 3 months of treatment. CLL monocytes were identified by staining with CD14 antibody. Viability of CD14+ cells was measured by Annexin V-PI analysis. Secretion of TNF-α was analyzed by CSA. Phagocytosis was tested using Zymosan A FITC-fluorescent BioParticles gating by flow cytometry CD14+/CD11b+cells.

### Statistical Analyses

Data were analyzed using SPSS version 20.0 (SPSS, Chicago, IL, United States). In some experiments, results were normalized on control (100%) (vehicle-treated samples). Normalization was performed by dividing the value of a particular sample treated with ibrutinib to the value of the corresponding sample treated with vehicle DMSO. *P-*values were calculated by Student *t*-test (*P < 0.05, **P < 0.01). Data are presented as mean and standard error of the mean (SEM) is depicted as error bars.

## Results

### Ibrutinib Modifies Gene Expression Profile of NLC

**FIGURE 1:**
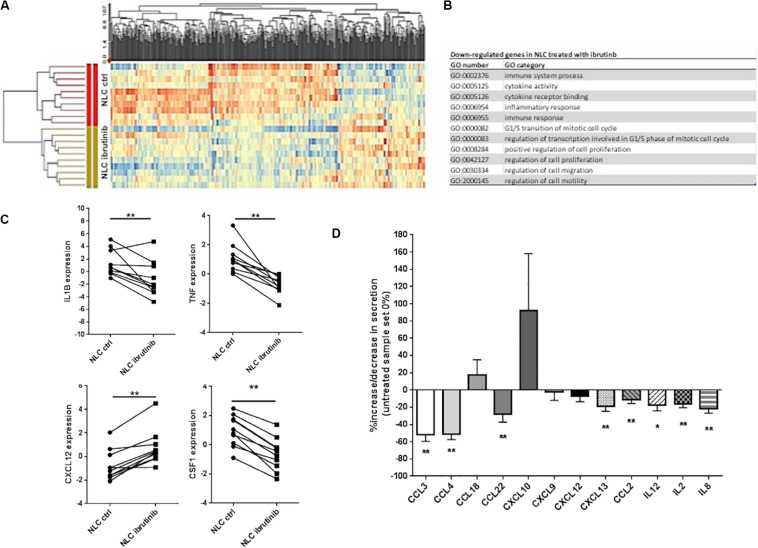
Ibrutinib modifies NLC gene expression profile. NLC were generated from PBMCs of 10 CLL patients after 15 days of culture. Then, CLL cells were carefully washed off by vigorously pipetting and adherent NLC were treated over-night with 1 μM ibrutinib or vehicle (DMSO). **(A)** Heat map depicts differentially expressed genes between NLC treated and not with ibrutinib. Genes were defined as differentially expressed between ibrutinib-treated vs. vehicle-treated group at a significant level of *P* < 0.05 and with a fold change cut off ±2. The supervised analysis identified 566 differentially expressed genes, 409 down-regulated and 157 up-regulated by treatment. **(B)** Among down-regulated genes, the most represented GO categories were related to immune system process, inflammatory response, immune response, cytokine activity, implying the ability of ibrutinib to modify the expression of genes implicated in immune function of NLC. **(C)** The down-regulated profile included several genes belonging to interleukin 1 (IL-1β) and tumor necrosis factor receptor family (TNF-α). Moreover, we found the down-regulation of several chemokine CXCL12 and CSF1. Values of untreated and treated samples (*n* = 10) are connected by lines (**P < 0.01). **(D)** Bar diagram depicts the level of chemokines and interleukins secreted by NLC either treated or not with ibrutinib. Secretion was measured on NLC supernatants by ELISA (*n* = 20, *P < 0.05, **P < 0.01).

To examine the molecular modifications induced by ibrutinib treatment, a large-scale GEP was performed on NLC generated from CLL patients after 15 days of culture. Treatment with ibrutinib affected neither NLC viability and nor morphology (data not shown). Genes were defined as differentially expressed between ibrutinib-treated vs. vehicle-treated group at a significant level of *P* < 0.05 and with a fold change cut off ±2. The supervised analysis identified 566 differentially expressed genes, 409 down-regulated and 157 up-regulated by treatment (Figure 1A). Among down-regulated genes, the most represented gene ontology categories were related to immune system process, inflammatory response, immune response, cytokine activity, implying the ability of ibrutinib to modify the expression of genes implicated in immune function of NLC (Figure 1B). In particular, the down-regulated profile included several genes belonging to tumor necrosis factor receptor family (TNF, TRAF1, TNFSF15, TNFRSF12A, TRAF4, TNFSF14, TNFRSF9) and interleukin 1 (IL1R2, IL1RN, IL1B) (Figure 1C, *n* = 10, *P* < 0.01). IL-1β is a potent pro-inflammatory cytokine that together with TNF-α plays a crucial role in host-defense responses to infection by bacteria, virus, parasites. TNF-α is a master regulator of inflammatory cytokine production triggering other molecules as IL-8, CCL3, CCL4, CCL2, MMP, ROS (25). On this line, our data from GEP showed a down-regulation of several chemokines, i.e., CCL1, CCL3, CCL7, CXCL13, CCL22, CCL4, and CSF1, and metallopeptidases. We detected an increased expression of CXCL12 (Figure 1C, *n* = 10, *P* < 0.01) that has a critical role in monocytes extravasation, enhances the expression of CD14 and CD163 and induces the secretion of angiogenic factors as VEGF and CCL1. Moreover, CXCL12 resulted up-regulated during inflammatory processes and cancer (26). In NLC, ibrutinib impaired the expression of CSF1, macrophage colony-stimulating factor, that activates *in vivo* and *in vitro* the anti-bacterial and anti-fungal activities of macrophages increasing the phagocytic activity and the production of reactive oxygen intermediates. In addition, CSF1 renders macrophages responsive to a secondary signal that triggers their immunological functions mediated by secretion of molecules as TNF-α (27). We analyzed the conditioned medium of NLC after treatment with ibrutinib. We detected a decrease in chemokines CCL3, CCL4, CCL22, CXCL9, CXCL12, CXCL13, and CCL2 and in interleukins IL12, IL2, and IL8 (Figure 1D, *n* = 17, *P < 0.05, **P < 0.01). Extremely variable levels of CXCL10 secretion were measured with about half NLC samples showing a huge increase, whereas others experiencing a decrease in its release. No modulation of the Th1 attractor CXCL9 and the naïve T cell attractor CXCL18 was detected (Figure 1D).

### BTK Inhibition Affects the Release of Pro-inflammatory Cytokines by Monocyte/Macrophage Population During Fungal Infection

**FIGURE 2:**
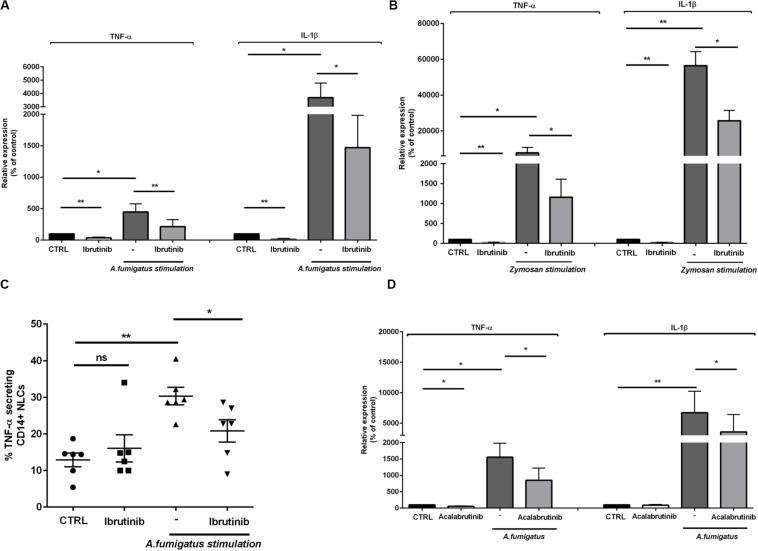
Ibrutinib alters the inflammatory profile of macrophage population. NLC were treated overnight with ibrutinib and then stimulated with *A. fumigatus* conidia or zymosan. **(A)** Bar diagrams show the expression level of TNF-α and IL-1β in NLC measured by real-time PCR. As shown ibrutinib decreased the amount of TNF-α and IL-1β in presence or not of *A. fumigatus* stimulation (*n* = 8, *P < 0.05, **P < 0.01). **(B)** Ibrutinib was able to reduce the expression of both TNF-α and IL-1β in NLC with or without zymosan stimulation (*n* = 8, *P < 0.05, **P < 0.01). **(C)** Dot plot diagram depicts the ability of NLC to secrete TNF-α after an overnight incubation with ibrutinib and then stimulated or not with *A. fumigatus* stimulation, measured by CSA. As shown, the level of TNF-α production after treatment with ibrutinib either in presence of *A. fumigatus* stimulation was significantly decreased (*n* = 6, *P < 0.05, **P < 0.01). **(D)** NLC were treated overnight with acalabrutinib and then stimulated with *A. fumigatus* conidia. Bar diagrams show the relative expression of TNF-α and IL-1β in NLC after treatment either in presence or not of stimulation (*n* = 6, *P < 0.05, **P < 0.01).

*Aspergillus fumigatus* induces the release of pro-inflammatory cytokines important for host defense as TNF-α and IL-1β. Considering that ibrutinib down-modulated TNF-α and IL-1β related genes in NLC, we focused our attention on their production. The expression of both TNF-α and IL-1β was accentuated by *A. fumigatus* stimulation and was negatively affected by blocking BTK (Figure 2A, *n* = 8, *P < 0.05, **P < 0.01). In addition, stimulation with zymosan intensified the expression of both TNF-α and IL-1β that was significantly limited by treatment with ibrutinib (Figure 2B, *n* = 7, *P < 0.05, **P < 0.01). Concordantly, secretion of TNF-α by NLC was strongly induced by pulsing the cells with *A. fumigatus* conidia (*P* < 0.01) and was significantly reduced by ibrutinib (Figure 2C, *n* = 6). To decipher the importance of BTK in NLC during a fungal infection, we inspected the effect of acalabrutinib, a more specific BTK inhibitor. Treatment of NLC with acalabrutinib affected the expression of TNF-α and IL-1β either without stimulus or in presence of *A. fumigatus* (Figure 2D, *n* = 7, *P < 0.05, **P < 0.01).

**FIGURE 3:**
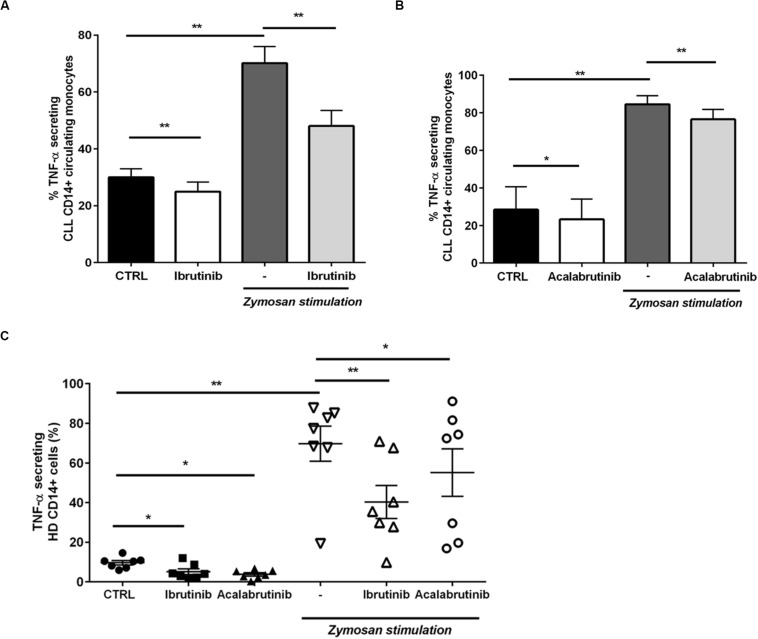
BTK inhibition modifies CD14+ circulating monocytes in CLL patients and healthy donor. **(A)** CD14+ monocytes were pre-treated with ibrutinib for 1 h and stimulated with zymosan. Bar diagrams show the secretion of TNF-α by CD14+ circulating population in CLL patients measured by CSA (*n* = 6, *P < 0.05, **P < 0.01). **(B)** CD14+ monocytes were pre-treated with acalabrutinib for 1 h and stimulated with zymosan. Bar diagrams show the secretion of TNF-α by CD14+ circulating population in CLL patients measured by CSA (*n* = 8, **P < 0.01). **(C)** Dot plots show the amount of TNF-α secretion in CD14+ monocytes in healthy donor volunteers. Ibrutinib and acalabrutinib strongly affected the level of TNF-α either in presence or absence of zymosan stimulation (*n* = 7, *P < 0.05, **P < 0.01).

Then, we moved toward analyzing the CD14+ circulating counterpart in CLL patients. Inhibition of BTK in monocytes by ibrutinib and acalabrutinib interfered with TNF-α secretion either in presence or absence of zymosan particles (Figures 3A,B, *n* = 6, *P < 0.05, **P < 0.01). Circulating CD14+ monocytes isolated from HD were tested during treatment either with ibrutinib or acalabrutinib and then stimulated with zymosan. Reduced levels of TNF-α secretion were detected after inhibition of BTK either in presence or absence of any stimulus. Monocytes responded to the addition of zymosan with high TNF-α secretion that was significantly counteracted by both ibrutinib and acalabrutinib (Figure 3C, *n* = 7, *P < 0.05, **P < 0.01).

### Ibrutinib Hampers NLC Response During *Aspergillus fumigatus* Infection

**FIGURE 4:**
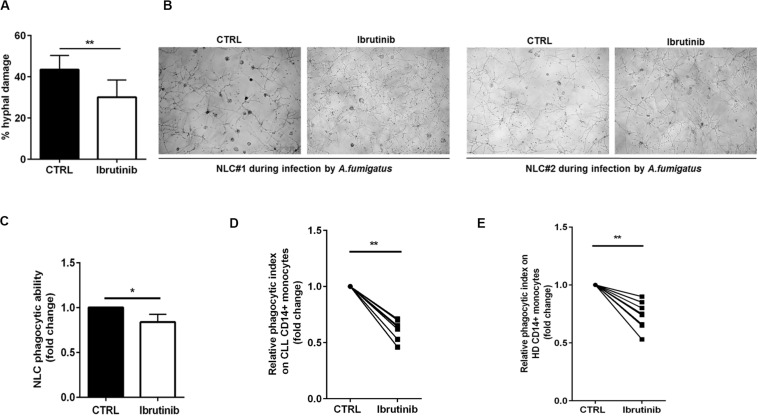
Ibrutinib affects the anti-fungal activities of NLC. **(A)** Bar diagram shows the percentage of hyphal damage induced by NLC either treated or not with ibrutinib (*n* = 5,**P < 0.01). **(B)** Photomicrographs show the growth of *A. fumigatus* onto NLC previously treated overnight with ibrutinib in two representative CLL patients. **(C)** NLC obtained from eight CLL patients were treated or not with ibrutinib overnight, followed by phagocytosis assay. Bar diagram depicts the reduction in phagocytic ability expressed in fold change induced by ibrutinib in NLC (*P < 0.05). **(D)** Circulating CD14+ CLL monocytes were pre-treated with ibrutinib for 1 h and then stimulated with zymosan particles labeled with a red dye. The phagocytic activity of monocytes was measured by flow cytometry gating CD11b+ CD14+ population. Ibrutinib significantly affected the ability of monocytes to engulf zymosan particles (*n* = 7, *P < 0.01). **(E)** CD14+ HD monocytes were pre-treated with ibrutinib for 1 h and then stimulated with zymosan particles labeled with a red dye. The phagocytic activity of monocytes was measured by flow cytometry (*n* = 9, **P < 0.01). Lines show the reduction in engulfment ability induced by ibrutinib.

A crucial step of *A. fumigatus* infection spreading is germination, i.e., the transition from resting conidia to invasive growth. Since ibrutinib accentuated an immunosuppressive profile of NLC, we determined its effect on NLC response to *A. fumigatus* infection. Firstly, we demonstrated the ability of NLC to induce damage in *A. fumigatus* hypha germination counteracting the fungal metabolic activity. Treatment with ibrutinib determined a reduction of this capability leading to a residual growth of *A. fumigatus* hyphae (Figure 4A, *n* = 5, **P < 0.01). In Figure 4B, two representative microphotographs show germination of *A. fumigatus* onto NLC culture. Then, we tested NLC treated with ibrutinib for their ability to phagocyte zymosan particles. As shown in Figure 4C, ibrutinib decreased the engulfment activity in 11 different NLC samples (*P < 0.05). To confirm this result, we analyzed the phagocytic function of CD14+ monocytes either in CLL patients or in HD. As shown in Figures 4D,E, ibrutinib significantly interfered with phagocytosis of zymosan both in CLL patients and in HD samples. A mean reduction of 40% in CLL monocytes (*n* = 7, **P < 0.01) and of 27% in HD monocytes (*n* = 9, **P < 0.01) was detected. Overall, our findings indicate that NLC-immune response is markedly inhibited by ibrutinib during *A. fumigatus* infection.

### Signaling Pathways Are Impaired by BTK Inhibition in NLC During Fungal Infection

**FIGURE 5:**
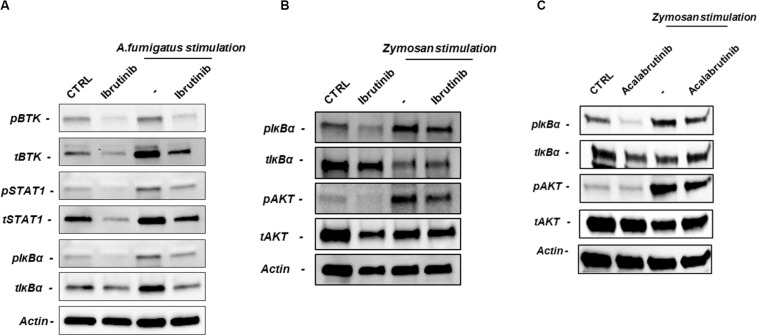
BTK inhibition impairs NLC–mediated response against *A. fumigatus* and zymosan. NLC were treated overnight with ibrutinib or acalabrutinib and then stimulated with *A. fumigatus* conidia for 2 h or with zymosan for 1 h. **(A)** Blots show the signaling pathways affected by ibrutinib during *A. fumigatus* stimulation. Ibrutinib efficiently inhibited phosphorylation of BTK, STAT1 and IκBα during conidia stimulation (*n* = 4). **(B)** Ibrutinib affects phosphorylation of AKT and IκBα during zymosan stimulation (*n* = 4). **(C)** NLC were treated with acalabrutinib overnight and then stimulated with zymosan for 1 h. Blots show a reduction of IκBα and AKT either in presence or absence of zymosan stimulation (*n* = 4).

In macrophages, BTK drives secretion of inflammatory cytokines upon TLR, NLRP3 and triggering receptor expressed on myeloid cells 1 (TREM-1) stimulation. On this line, we asked if inhibition of BTK through ibrutinib and acalabrutinib was able to counteract the activation of inflammatory signaling pathways by swollen *A. fumigatus* conidia or zymosan stimulation. Ibrutinib blocking BTK phosphorylation interfered with STAT1 and IκBα phosphorylation during conidia stimulation (Figure 5A, *n* = 4). Accordingly, during zymosan stimulation ibrutinib decreased the level of IκBα and AKT phosphorylation (Figure 5B, *n* = 4). We also tested the effects of acalabrutinib on NLC during zymosan stimulation detecting a significant inhibition in IκBα and AKT phosphorylation (Figure 5C, *n* = 4). Of note, we also detected a reduction of total amount of BTK, STAT1, and IκBα.

### Monocytes Show an Impairment of Immunomodulatory Features During Treatment With Ibrutinib in CLL Patients

**FIGURE 6:**
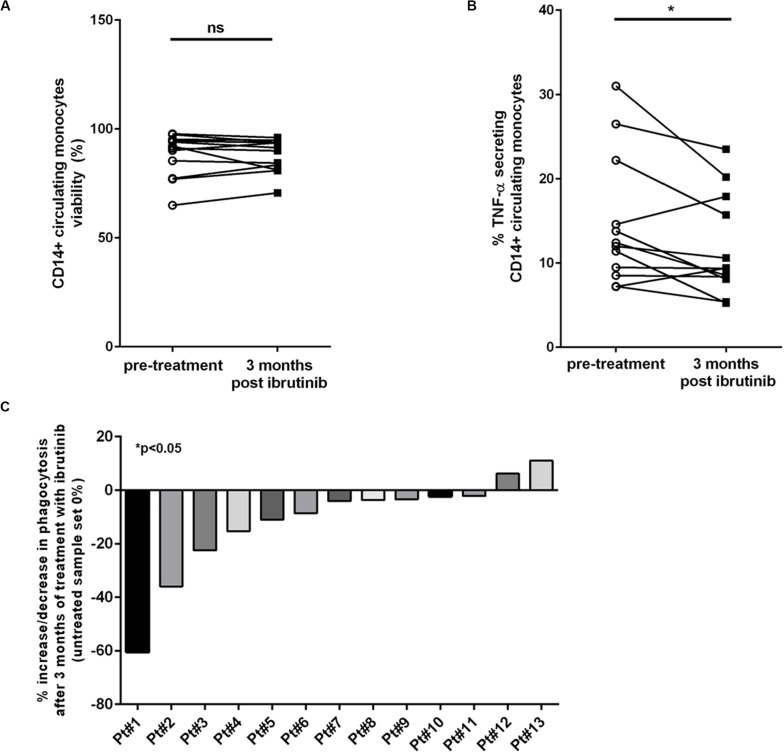
Circulating CD14+ monocytes are altered during ibrutinib treatment in CLL patients. Blood samples isolated from CLL patients pre-treatment and after 3 month of treatment with ibrutinib were analyzed. **(A)** Diagram shows the viability of CD14+ monocyte population. Ibrutinib did not affect the survival of monocytic population (*n* = 14, *P* = ns). **(B)** Secretion of TNF-α was measured in 12 CLL patients. Diagram shows a significant reduction of TNF-α levels after 3 months of treatment with ibrutinib (*n* = 12, *P < 0.05). **(C)** The phagocytic activity was measured in 13 CLL samples. Bar diagram shows the percentage of increase or reduction in phagocytic activity after 3 months of treatment with ibrutinib compared with pre-treated sample for each patient (*n* = 13, *P < 0.05).

Different studies have demonstrated insurgence of early-onset IA and other fungal infections in patients treated with ibrutinib (14). Given this occurrence of infections, we planned to analyzed blood samples isolated from CLL patients during treatment with ibrutinib comparing the CD14+ monocytic population before treatment and after 3 months (Supplementary Table 3). Firstly, we measured the viability of CD14+ circulating cells observing no difference between the two time-points (Figure 6A, *n* = 14, *P* = ns). Then, we focused the attention on the immunological properties of monocytes before and during treatment with ibrutinib. We analyzed the ability of monocytes to release TNF-α. As shown in Figure 6B, we monitored a significant decreased of TNF-α secretion after 3 months of treatment (*n* = 12, *P < 0.05). A mean secretion of 14.7% (±2.2%) in pre-treatment samples was reduced to 11.8% (±1.7%) during the first 3 months of therapy. Among the CLL samples, 5 of 12 experienced an important drop in TNF-α secretion during treatment, instead we detected a slight increase in just two samples. We examined the ability of CD14+ cells to engulf zymosan particles. As reported in Figure 6C, we found a significant reduction of phagocytosis ranging from a decrease of 60 to 2% (*n* = 13, *P < 0.05) with just two CLL patients showing an opposite trend with an increase of 6 and 11%, respectively. Altogether, these results reported for the first time the biological effects induced in the monocytic population of CLL patients during treatment with ibrutinib, highlighting how inhibition of BTK affects its inflammatory profile that may compromise response to fungi infection.

## Discussion

In this study, we report how BTK inhibition compromises a productive inflammatory response by monocytes in CLL patients during *A. fumigatus* infection. Ibrutinib is a first-in-class irreversible BTK inhibitor affecting pathways downstream of BCR in malignant B lymphocytes (28), but has also potent effects upon the normal cells of the immune system executing an immunomodulatory effect. Ibrutinib targets BTK expressed by CLL-associated macrophages (also known as nurse-like cells, NLC) accentuating their immunosuppressive profile through up-regulation of M2 polarization markers and impairing the phagocytic activity (11). Although, in CLL, ibrutinib has proven to be efficacious in relapse/refractory patients and in high risk patients with TP53 deletion or mutation (29, 30) resistance and important adverse effects have been reported over the years. Invasive fungal infections (IFIs) have been reported among patients receiving treatment with ibrutinib. An unexpected high incidence of Pneumocystis jirovecii pneumonia has been described in CLL patients receiving single-agent ibrutinib (31). In addition cases of IA have been characterized by an early onset, mild clinical manifestations, asymptomatic/low symptomatic pulmonary localization, and high risk of CNS involvement (14, 16, 32, 33). The reason why an association of IFI during treatment with ibrutinib was observed is still not completely clear. Given the observation that the occurrence of IFI are rare in CLL, it is evident that some patients are characterized by variable immunodeficiency and ibrutinib may act on the immunological mechanisms implicated in a productive fungal response. On these evidence, our study sought to determine the immunological response that characterize the specific CLL-monocyte and macrophage population in order to dissect their involvement during an infection by *A. fumigatus*. The infectious life cycle of *A. fumigatus* starts with the production of conidia (asexual spores) that are dispersed in the environment. The primary route of infection is via inhalation of conidia followed by conidia deposition (1). The first line of defense is represented by phagocytic process, secretion of microbiocidals and regulation of adaptive immune system. Macrophages phagocytose *A. fumigatus* conidia in an actin-dependent manner, a process mediated by the recognition of pathogen-associated molecular patters (PAMP) by host PRRs (pattern recognition receptors) (1). The first question we asked ourselves in this perspective concerned the potential effects of ibrutinib to influence the ability of macrophage in CLL to effectively obstruct the growth of *A. fumigatus*. Conflicting results of ibrutinib effects on phagocytic ability have been published over the years. Several studies have demonstrated the involvement of BTK in FcγR-mediated phagocytosis in macrophages. Activation of BTK is implicated during ingestion and also when phagosomes are mature (34). Phagocytosis of *A. fumigatus* conidia activates TLR9 recruitment to the phagosome and, through BTK, induces phospholipase C-gamma and calcineurin mediated NFAT nuclear translocation (13). Recently, Bercusson et al. (24) reported an impairment in fungal growth without detection of significant difference in phagocytic activity of human-monocyte-derived macrophages treated with ibrutinib, in addition a reduction of FcγR-mediated cytokine production, but not phagocytic ability was seen in human blood monocytes (35). Oppositely, BTK-deficient macrophages show defects in fungal phagocytosis and TLR-mediated phagocytosis of tumor cells seems to be impaired by ibrutinib treatment of macrophages, implying a relevant role of BTK in the phagocytic activity of innate immune cells (36, 37). These results were also confirmed in neutrophil population with a reduction of *A. fumigatus* engulfment and killing in patients receiving ibrutinib (14). Recently, we have demonstrated an impairment of phagocytic activity in NLC together with a significant reduction of MAC-1 (CD11b/CD18) activation, essential for the phagocytic cup formation (11). Here, in this context ibrutinib affected the ability of NLC to counteract *A. fumigatus* conidia germination and we linked this inability to a reduced phagocytosis. To confirm this result, we also tested the circulating monocytic population isolated both from CLL patients and also from HD detecting a significant decreased of phagocytosis upon ibrutinib treatment. Fungal cell wall contains polysaccharide and lipid moieties that activate an immune response with a strong production of cytokines including TNF-α, IL1β, IL-6, IL-8. The characterized PRR for *A. fumigatus* include β-glucan receptor Dectin-1, the CD11b/CD18 (complement receptor 3, CR3), the TREM-1 and TLRs. BTK represents a crucial molecule in the transmission of signaling cascade from all these immune receptors (3). Our aim was to outline the impairment in the inflammatory pathways caused by ibrutinib during infection by *A. fumigatus* conidia, or more generally by using zymosan. Stimulation of NLC with *A. fumigatus* conidia or zymosan induce the phosphorylation of BTK which in turn activate downstream cascade with STAT1, IκBα, and AKT. As observed by Cervantes-Gomez et al., ibrutinib therapy determined a decrease in phospho- as well as total BTK protein in CLL lymphocytes and a decline in BTK total protein was observed also in circulating leukemic cells after 4 weeks of treatment (38, 39). In our setting, exposure to ibrutinib determined a reduction in the activation of these pathways on CLL-derived macrophage that confirmed a previous work on human macrophages isolated from HDs (24). Further signaling studies, with and without zymosan depleted Saccharomyces cell wall, are needed to measure the involvement of TLR mediated signaling. Since PRR engagement is able to determine phagocytosis, macrophage activation and a strong induction of pro-inflammatory response, we aimed to determine the inflammatory profile of NLC and CLL monocytes during stimulation with *A. fumigatus* and zymosan. Ibrutinib intensely forced an immunosuppressive profile in NLC. Our data from gene expression profile showed a relevant impairment in the expression of genes related to TNF and IL1 family. Of note, expression and secretion of TNF-α and IL-1β in NLC were strongly affected by ibrutinib mimicking fungal infection. Confirmation of a reduction in TNF-α secretion ability was also found in CD14+ CLL monocytes and in HD monocytes. Since until today there are not definitive data about fungal infections during acalabrutinib treatment, we argue to determine the biological effects of acalabrutinib on the monocytes/macrophage counterpart in CLL. Acalabrutinib is a second generation, potent BTK inhibitor, more selective for BTK than ibrutinib and therefore has the potential for fewer off-targets adverse events showing high response rate and durable remissions (29). To our knowledge as far as acalabrutinib is concerned the rate of infection varied in different preliminary reports, and only one case of fungal infection after 2 months of treatment was reported in phase 1–2 clinical trials (21, 22, 40). As demonstrated with ibrutinib, in NLC acalabrutinib affected signaling cascade during zymosan activation and also the expression of TNF-α and IL-1β The reduction of TNF-α induced by acalabrutinib was also detected in CLL circulating monocytes and HD donors upon fungi stimulation. Altogether our results showed the effects of ibrutinib and acalabrutinib in modulating CLL-derived monocytes and tissue macrophages features during *A. fumigatus* infection, underlying the importance of BTK expression as a “guardian” of the innate immunity. This information has to be considered in this new therapeutic era, because the best-characterized BTK inhibitors, ibrutinib and acalabrutinib, are employed every day in the clinical practice thanks to consistent results, but at the same time present some adverse effects.

Several evidence have shown an impairment of the innate but not of the adaptive immunity during treatment with ibrutinib (41). Patients with lymphoid cancer receiving treatment with ibrutinib are at risk of infection, including IFI. The frequencies of IFI is identical comparing patients who received ibrutinib as first-line and those who had received more than one treatment. This may imply that ibrutinib may drive infection risk rather than prior treatment (42). In this scenario, our second question was related to the biological modifications on monocyte population that may be caused during treatment with ibrutinib. As known, ibrutinib is becoming a standard of care in the treatment of CLL, however IFIs have been reported. Although an *in vitro* system can help to understand the involvement of the immune system during a fungal infection; it cannot recapitulate the dense and complex network of crosstalk between the leukemic cells and accessory cells, so our attention was focused on the biological effects in CLL patients undergoing to treatment with ibrutinib. In this scenario, every patient may be considered unique given its individual immunological features related to the disease. Given the observation that the occurrence of IFI are rare in CLL, it is evident that some patients are characterized by variable immunodeficiency and ibrutinib may act on the immunological mechanisms implicated in a productive fungal response. We aimed to analyze circulating monocytes before and after 3 months of treatment with ibrutinib. Of note, most cases of IFI occurred with a median of 3 months after starting ibrutinib. In our cohort of CLL patients, we found confirmation of our *in vitro* data. Basal secretion of TNF-α by circulating monocytes was effectively reduced after initiating ibrutinib therapy. In addition the ability to phagocytose was counteracted during treatment with ibrutinib. As previously reported by us, ibrutinib, targeting BTK expressed by monocytes, confers an exacerbation of some immunosuppressive features. From this perspective, these modifications may compromise an efficient inflammatory response during *A. fumigatus* infection leading to a possible insurgence of IFI. Unfortunately, we have not the possibility to test samples isolated from CLL patients treated with acalabrutinib. Future investigation is warranted to explore the influence of acalabrutinib and zanubrutinib on the CLL monocytic population.

## Conclusion

In conclusion, our study offers a new brick in the knowledge of ibrutinib and acalabrutinib treatment consequences on the immune system in CLL patients. Moving from “bench” to “bedside” and back again, clinicians are now warned to the importance of the multiple biological consequence that a single player, as BTK, may have also in the clinical practice. Further work is required to understand why some patients are more prone to fungal infections during treatment to identify who might benefit from prophylaxis and to define factors that add to the risk.

## Data Availability Statement

The datasets presented in this study can be found in online repositories. The names of the repository/repositories and accession number(s) can be found below: https://www.ncbi.nlm.nih.gov/geo/, GSE142292.

## Ethics Statement

The studies involving human participants were reviewed and approved by the Comitato etico proviciale. Azienda Ospedaliero-Universita di Modena pratica 187/15. The patients/participants provided their written informed consent to participate in this study.

## Author Contributions

SF and RMf conceived and coordinated the research, and interpreted the results. SF analyzed the results, wrote the manuscript, performed the *in vitro* experiments, acquired and analyzed flow cytometric data, and performed the statistical analyses. RMf performed some molecular analyses, supervised the work-flow, and revised the results and the manuscript critically. LS and MM provided samples for flow cytometry evaluation. LP, PG, ML, and RMr revised and approved the final version of the manuscript. All authors contributed to the article and approved the submitted version.

## Conflict of Interest

The authors declare that the research was conducted in the absence of any commercial or financial relationships that could be construed as a potential conflict of interest.

## Footnotes

1 http://www.geneontology.org/

2 https://www.ncbi.nlm.nih.gov/geo/

## Abbreviations

Presented in abstract form at the 24th annual meeting of the European Hematology Society: Amsterdam June 2019.
