# Supplementary material for: BTK Inhibition Impairs the Innate Response Against Fungal Infection in Patients With Chronic Lymphocytic Leukemia
Source: Front Immunol. 2020 Aug 28;11:2158. doi: 10.3389/fimmu.2020.02158 (PMC7485008; doi:10.3389/fimmu.2020.02158)
Supplement: Supplementary file 1 [file Data_Sheet_1.doc]

**Supplementary Table 1.** Primers used in real-time PCR

| **Gene** | **Forward primer** | **Reverse primer** |
| --- | --- | --- |
| **TNF-α** | 5’-CAGCCTCTTCTCCTTCCTGAT-3’ | 5’-GCCAGAGGGCTGATTAGAGA-3’ |
| **IL-1β** | 5’-TACCTGTCCTGCGTGTTGAA-3’ | 5’-TCTTTGGGTAATTTTTGGGATCT-3’ |

**Supplementary Table 2.** Antibodies used in immunoblotting

| **Antibody** | **Manufacturer** |
| --- | --- |
| **Anti-Akt rabbit** | Cell Signaling Tech, Beverly, MA |
| **Anti-phospho-Akt rabbit** | Cell Signaling Tech, Beverly, MA |
| **Anti-BTK rabbit** | Cell Signaling tech, Beverly, MA |
| **Anti-phospho-BTK rabbit**  **Anti-phospho-IĸBα**  **Anti-IĸBα** | Cell Signaling tech, Beverly, MA  Cell Signaling tech, Beverly, MA  Cell Signaling tech, Beverly, MA |
| **Anti-phospho-STAT1 mouse**  **Anti-STAT1 mouse**  **Anti-βactin mouse** | ECM Biosciences, Versailles, KY  Cell Signaling Tech, Beverly, MA  Abcam, Cambridge, UK |

**Supplementary Table 3**. Characteristic of patients and blood samples included in the study

| **Characteristics** | **Before ibrutinib therapy** | **Month 3** |
| --- | --- | --- |
| Numbers of patients | 14 | 14 |
| Mean age in years (range) | 70.5 [41-84] | 70.5 [41-84] |
| Male/female sex | 10/14 | 4/14 |
| Dosage of ibrutinib (mg/per day) | Not applicable | 420 [14/14] |
| Number of theraperutic line  before ibrutinib: mean (range) | 1.1 [1-3] | 1.1 [1-3] |
| Infectious complications (yes or no) (range) | Not applicable | Yes [6/14]  No [8/14] |

**Patients and samples**

Peripheral blood mononuclear cells (PBMCs) were isolated by Ficoll density gradient centrifugation and used fresh or cryopreserved in RPMI-1640 medium (Life Technologies, Carlsbad, CA, USA), 50% fetal bovine serum (FBS), and 10% dimethyl sulfoxide (DMSO) and stored in liquid nitrogen until use. To generate NLCs, PBMCs from CLL patients were cultured (10^7^/mL) in RPMI-1640 medium with 10% FBS, 50µg/mL gentamicin, 100U/mL penicillin, 100µg/mL streptomycin for 10 days. Fresh medium was added to the culture every 3 days.

**Analysis of gene expression profile**

CLL cells were carefully washed off and adherent NLCs were treated over-night with 1µM ibrutinib or vehicle. NLCs were lysed to obtain RNA samples. Total RNA was extracted by using RNeasy Mini kit Plus (QIAGEN). Large-scale gene expression profiling (GEP) was performed by hybridizing RNA on 4X44K Whole Human Genome Microarray (Agilent Technologies). Fluorescence data were analyzed with Feature Extraction Software v10.5 (Agilent Technologies). Supervised analysis based on paired t-test with multiple testing correction (Benjamini Hochberg FDR) were performed by using Gene Spring GX v11.5 (Agilent) software. Genes were defined as differentially expressed between ibrutinib-treated vs. vehicle-treated group at a significant level of p<0.05 and with a fold change cut off ± 2. Gene Ontology Tool (http://www.geneontology.org/) was used to classify genes in functional categories. Data have been deposited in NCBIs Gene Expression Omnibus (GEO, https://www.ncbi.nlm.nih.gov/geo/ , GSE142292).

**Real time PCR**

RNA was extracted with the RNeasy Plus Mini kit (Qiagen, Valencia, CA, USA). RNA (100ng) was reverse transcribed using Transcription High fidelity cDNA Synthesis kit (Roche Applied Science, Penzeberg, Germany). All samples were analyzed in real time on LightCycler 480v.2 (Roche) in duplicate). Amplification of the sequence of interest was normalized to an housekeeping reference gene (Glyceraldehyde 3-phosphate dehydrogenase, GAPDH) and compared to a calibrator sample (Universal Human Reference RNA; Stratagene, Cedar Creek, TX).

**XTT assay**

NLCs were cultured in 96 well plate and then treated with ibrutinib or DMSO. Conidia of *Aspergillus fumigatus* (2×10^3^ per well) were plated over NLC or alone (positive control) and incubated at 37°C for 36 hours to allow germination. Each experimental condition was performed in triplicate. To characterize the lytic activity of NLC against *Aspergillus fumigatus*, a colorimetric assay with (2,3-bis[2-methoxy-4-nitro-5-sulfophenyl]2H-tetrazolium-5-carboxyanilide) sodium salt (XTT; Sigma) plus coenzyme Q0 (2,3-dimethoxy-5-methyl-1,4-benzoquinone; Sigma) was used. Anti-hyphal activity was expressed normalizing the absorbance of experimental wells (CTRL vs ibrutinib) with NLC to the absorbance of wells with hyphae only X 100.

**NLC phagocytosis assay**

Phagocytosis was inspected by using CytoSelect^TM^ 96-Well Phagocytosis assay (Cell Biolabs, San Diego, CA, USA) according to manufacturer’s instructions. NLCs were generated from CLL patients and then treated with ibrutinib 1 hour. After incubation, zymosan particles were added to cells for 1 hour, then NLCs were fixed and external zymosan particles were blocked. After permeabilization, zymosan particles engulfed by NLCs were measured by colorimetric detection.

**Cytokine secretion assay (CSA)**

NLCs or PBMCs isolated from CLL patients or healthy donors were treated with ibrutinib or acalabrutinib for 24 hours and stimulated with germinated boiled killed *A.fumigatus* inactivated conidia or zymosan and analyzed using CSA for TNF-α according to manufacturer’s instructions (CSA Detection kit; Miltenyi Biotec). Cells were immunostained with TNF-α catch reagent and incubated for 2 hours at 37°C to allow cytokine secretion. After washes, cells were labeled with TNF-α Detection antibody conjugated to PE and CD14 APC Ab. An isotype control sample for each condition was acquired to exclude autofluorescence background.

**Immunoblotting**

Proteins (80ug/lane) were electrophoresed on 4% to 20% SDS-polyacrylamide gradient gels (Biorad laboratories, Hercules, CA, USA). Membranes were immunoblotted with primary antibodies listed in Supplementary Table 2 and incubated with species-specific horseradish peroxidase (HRP)-conjugated secondary antibody (diluted 1:50000; GE Healthcare, Uppsala, Sweden) for 1 hour and developed using HRP conjugates Western Bright Sirius (Advasta, Menlo Park, CA, USA). Images were acquired and analyzed using Image Lab Software v.3.0 (Biorad Laboratories). NLCs were pretreated with ibrutinib or acalabrutinib overnight following to stimulation with 2x10^5^/ml of germinated boiled killed A.fumigatus inactivated conidia for 2h or 50 μg/ml of zymosan for 1h.

**CD14+ monocytes phagocytosis assay**

PBMCs isolated from CLL patients or healthy donors were suspended in culture medium either in presence or absence of ibrutinib for 1h. Zymosan A FITC-fluorescent BioParticles (Molecular probes, Eugene, Oregon) were added and incubated with cells at 37°C for 1h. Then, PBMCs were stained with CD14 APC and CD11b PE Abs. To distinguish the cells which have phagocytosed these from those simply binding the beads at the surface, a short incubation with trypan blue, followed by a wash with PBS, quenched surface FITC fluorescence. Analysis was performed by flow cytometry gating CD14+/CD11b+ cells and analyzing the mean fluorescence intensity in the positive zymosan population.
